# Development and validation of the housing instability scale

**DOI:** 10.1080/10530789.2022.2127852

**Published:** 2022-09-30

**Authors:** Adam Farero, Cris M. Sullivan, Gabriela López-Zerón, Ryan P. Bowles, Mackenzie Sprecher, Danielle Chiaramonte, Jasmine Engleton

**Affiliations:** a Department of Psychology, University of Michigan, Ann Arbor, MI, USA;; b Department of Psychology, Michigan State University, East Lansing, MI, USA;; c Department of Human Development and Family Studies, University of Michigan, Ann Arbor, MI, USA;; d Yale School of Public Health, Department of Social and Behavioral Sciences, Yale University, New Haven, CT, USA

**Keywords:** homelessness, housing instability, measurement, scale development, Spanish speakers

## Abstract

Despite increasing attention to the importance of examining factors that impact housing instability and homelessness, the field lacks a validated scale of housing instability. The current study examined the reliability and validity of a seven-item scale that measures housing instability. Data were taken from a larger study which implemented the Domestic Violence Housing First model across five domestic violence agencies in the Pacific Northwest. A total of 406 participants were interviewed every six months over a period of two years. A Spanish version of the scale was administered to Spanish-speaking participants. Results provide an overview of the psychometric functioning of the scale and support its utility in assessing housing instability and homelessness. Specifically, the scale demonstrated concurrent and predictive validity, and showed evidence of scalar equivalence over time and across both language and locality. The current scale is therefore a succinct and psychometrically sound measure of housing instability which can be used moving forward to track housing instability in English and Spanish speakers, as well as in urban and rural settings.

## Introduction

Housing instability is a pervasive and devastating problem in the United States, impacting millions of people. In 2017, 37.8 million people experienced housing-cost-burden, with 18.2 million severely housing cost-burdened – spending 50 % or more of their income on housing (Joint Center for Housing Studies of Harvard University, 2019). Additionally, in 2020, over half a million people were homeless, with approximately 350,000 of these individuals experiencing sheltered homelessness (i.e. living in emergency shelters or transitional housing programs; United States, 2020). Such statistics are especially concerning considering that both housing instability and homelessness are associated with a variety of adverse outcomes (Kang, 2021; Levitt et al., 2009).

Despite the enormity of this problem, and an increasing amount of policy and research aimed at studying its antecedents and sequelae, housing instability can be a challenge to consistently define and measure (Frederick et al., 2014; Kushel et al., 2006). For the purposes of identifying individuals in need of support, as well as for evaluating the effectiveness of housing interventions and other social services, it is critical to be able to measure housing instability in a way that is straightforward and user-friendly. The current study focused on creating and validating a scale of housing instability that would be brief, easily administered, and psychometrically sounds.

### Current definitions and measures of housing instability

One likely reason the field has lacked a cogent measure of housing instability is that there is no standard definition of the construct. Some prior studies have defined housing stability as simply having continuous housing for six months (e.g. Dickson-Gomez et al., 2008; North et al., 2010) while other single-item measures of housing instability have included “two or more moves in the prior year,” “being worried about losing housing or not having enough money for housing,” “having a lease violation,” or “number of evictions” (Adams et al., 2021; Breiding et al., 2017; Brisson & Covert, 2015; Dichter et al., 2017; Ma, Gee, & Kushel, 2008; Montgomery et al., 2018; Phinney et al., 2007; Reed et al., 2011).

While the number of moves within a brief period of time is one reasonable indicator of housing instability, and indeed, has been found to relate to having lower income, household instability (e.g. change in the number of individuals living together), and mental health disorders such as depression and general anxiety disorder, it is not sensitive enough as a single-item to determine housing instability (Adams et al., 2021; Desmond & Perkins, 2016; Suglia et al., 2011). Moving is not always a negative event and may be indicative of a more transient stage of life, such as in young adulthood, or may even be an indicator of moving into more stable housing (Collins & Curtis, 2011).

Other researchers examining homelessness or housing instability have created brief measures in order to test their hypotheses, but have not provided strong psychometric evidence for them. One was created specifically to assess the risk of homelessness among veterans (Montgomery et al., 2014), and two studies (De Marchis et al., 2021; Sandel et al., 2018) determined housing instability as positive endorsement of three items: being behind on rent, having moved more than two times in the prior year, and being currently homeless. Other scholars have classified different housing situations into categories that they determined to be more or less stable (e.g. with renting is considered to be stable, and staying with friends is considered unstable), but there has been inconsistency among these categories as well (e.g. Eastwood & Birnbaum, 2007; Tsemberis et al., 2007; Vijayaraghavan et al., 2011). Further, there can be vast differences in the level of stability within each of these housing categories (Collins & Curtis, 2011). Someone may be renting, for example, while also being in the process of being evicted. Another person may be staying with family and not paying rent, but that could in reality be a stable situation benefitting the entire family.

Four measures of housing instability were especially relevant to our purposes and guided the development of our scale. The residential timeline follow-back inventory (TLFB; New Hampshire Dartmouth Psychiatric Research Center, 1995) asks participants to work backwards over time to denote housing types, locations, and transitions. Although the TLFB is quite comprehensive and has demonstrated strong psychometric properties (Tsemberis et al., 2007), it is extremely time-consuming to administer, so it would not be easily incorporated into studies measuring additional constructs. Relatedly, the TLFB offers a variety of different ways to compile information from the measure into housing stability variables (e.g. number of moves, proportion of time stably housed). As a result, coding TLFB data can be time-consuming and messy depending on each participant’s housing history. Further, the aggregate variables produced from the TLFB may differ depending on the researcher’s broader definition of housing instability and homelessness.

Routhier (2019) constructed and tested a 10-item index that measures housing insecurity within four dimensions (unaffordability, poor living conditions, overcrowding and forced moves) using data from the United States Department of Housing and Urban Development’s (HUD) American Housing Survey (AHS). Analysis showed that, among the four dimensions, unaffordability was the most salient, followed by poor living conditions, forced moves, and crowding. This index was used in recent studies examining housing insecurity, including in minority and adolescent populations (Marçal et al., 2022; Merritt & Farnworth, 2021). Though this index was tested and includes important dimensions of housing instability, the authors discussed important limitations with certain variables, such as the tendency for overcrowding to be underreported (Routhier, 2019). In addition, the index only reflects the housing conditions of renters, excluding people who are unstably housed but not renting.

In another relevant housing study, Frederick et al. (2014) conducted qualitative interviews with 51 formerly homeless youth as a means of identifying the most important dimensions of housing stability to measure. They found that the most salient elements to include for youth are housing type, recent housing history, current housing tenure, financial status, involvement with the legal system, education level, employment status, harmful substance use, and subjective assessments of housing satisfaction and stability. Based on these finding, Frederick et al. (2014) created the 13-item Housing Security Scale. However, this is a scale of housing security, which is different from housing stability, and their scale has yet to be validated.

A final measure that influenced the development of our scale is the 10-item Housing Instability Index (Rollins et al., 2012), developed within a longitudinal study of unstably housed domestic violence (DV) survivors. That scale has also not been formally validated and includes four items that are only relevant to individuals with landlords. It does have promising face validity and includes an item measuring the number of moves but does not consider homelessness in its assessment of housing instability.

No brief measure of housing instability can be all-inclusive nor comprehensive, and all will have their advantages and disadvantages. A particular disadvantage across all four of these measures is that they have not been validated with Spanish-speaking populations. Homeless Latinx individuals experience unique disparities when it comes to receiving services, and as such require more culturally-specific support (Chinchilla & Gabrielian, 2020). Additionally, existing housing instability literature tends to focus on urban populations (e.g. Bottino et al., 2019; Carrion et al., 2015; Desmond et al., 2015; Routhier, 2019). The studies that do address housing instability in rural settings often focus on veterans and youth (Albright et al., 2022; Byrne et al., 2020; Curry et al., 2020; O’Brien et al., 2021). The current study, therefore, focused on developing and validating a brief scale that could adequately differentiate individuals based on housing instability across English-speaking and Spanish-speaking populations, as well as across urban and rural settings.

## Methods

The Housing Instability Scale (HIS) was created and validated within a larger, longitudinal study of domestic violence (DV) survivors. The parent study involved testing the effectiveness of a housing-focused intervention for unstably housed DV survivors (Domestic Violence Housing First; DVHF) over a period of two years. Although the study intentionally enrolled 400 unstably housed DV survivors in order to test the DVHF model, we anticipated that participants’ level of housing instability would change across time. Therefore, we conceptualized housing instability as a construct that would capture the precariousness of participants’ housing situation over a period of time (e.g. the past or upcoming six months), rather than as a snapshot of the state of their current housing situation. We designed the HIS to capture a variety of aspects of housing instability, including a six-month history of homelessness and the number of moves (see Table 1 for all items). This means that the current study did not distinguish between being homeless and unstably housed, but instead considered a history of homelessness in the last six months as an indicator of greater housing instability. Participants were interviewed every six months over a two-year time span, allowing us to examine the time sensitivity and predictive ability of the newly-created HIS.

### Participants

Study participants were recruited from five DV agencies in a state in the Pacific Northwest. Survivors were approached about the study shortly after they entered services, and eligibility included being a recent adult survivor of DV and being homeless or at imminent risk of becoming homeless. The study enrolled 406 participants who met the eligibility criteria and who agreed to be interviewed every six months over two years (baseline, 6-months, 12-months, 18-months, 24-months). Interviews were conducted in English (n = 358; 88%) or Spanish (n = 48; 12%), depending on participant preference. Survivors were paid $50 per interview, and all interviews were conducted either in-person or over the phone (based on participant preference) in safe and confidential locations. Participant retention rate remained high throughout the study. At 24 months, 89% of participants were retained. Human subjects approval was obtained for this study through the second author’s university. More information about the larger study can be found at (Sullivan et al., 2020).

### Sample

Study participants were predominately female (97%) and heterosexual (86%). Ages ranged from 19 to 62 years, with an average of 34.5 (*SD* = 9.02, *MDN* = 34) years old. Thirty-five percent were non-Hispanic white and 65% reported a racial/ethnic minority identity. Most participants (74%) had minor children they were responsible for. Over half of the participants had been employed (58%) at some point in the six months prior to participating in the study, but only 35% were employed at study entry. Of those who had lost their jobs in the prior six months, 70% reported it was due to the abuse they had experienced.

At study entry, 42% of the participants were homeless (36% living in a shelter, and 6 % unsheltered homeless). The other participants were unstably housed: 24% were in homes they owned or were renting, 22% were staying with family and friends without paying rent, 9 % were living with family and friends and paying part of the rent, and 3 % were in transitional housing or a drug treatment program.

Most study participants (73%) had a prior history of homelessness. Of those who had been homeless, the average cumulative amount of time spent homeless was just over two years. About one-third of those with a history of homelessness (33%) had been homeless at least once before age 18. Most of the sample (87%) had stayed with family or friends at least once in their life to avoid homelessness.

The primary language for most survivors was English (80%), followed by Spanish (15%). Immigrant survivors represented 18% of participants. The highest educational level attained by participants varied considerably. Twenty-nine percent had not completed high school, 22% had a high school diploma/GED, 29% had some vocational training or had attended college classes, and 20% had college degrees (either Associate’s, Bachelor’s or advanced degrees).

### Intentional focus on Latinx study participants

The study design included an intentional focus on recruiting and retaining Latinx participants, as too little research has focused on their experiences with DV and housing instability. All of our efforts were grounded within a Language Justice framework (Antena Aire, 2020), which centers study participants’ language needs and preferences throughout the research process (López-Zerón et al., 2021). Recruitment and data collection materials were carefully translated and reviewed by a bilingual and bicultural team led by the third author, who is a Latina immigrant and native Spanish-speaking researcher. As the Latina survivors from the study’s geographic area were predominantly from Mexico and Central America, we ensured that the language used in the translated materials was culturally and contextually relevant to this population.

Additionally, we hired bilingual and bicultural data collectors from the communities in which we were collecting data, who were well acquainted with the experiences of immigrant Latinx communities. Bilingual and bicultural staffs were trained in both English and Spanish to ensure they had a deep understanding of the instrument in both languages. Training in both languages also allowed for language-specific nuances to be clarified, and offered interviewers the opportunity to refine their interviewing skills. Ongoing supervision in both languages also ensured that data were being collected accurately. Further, our data management procedures included a rigorous data verification process, in which bilingual and bicultural data team members listened to audio recordings of interviews in English and Spanish to confirm the accuracy of the data.

Finally, we are committed to validating study measures in Spanish if they have not already been adequately validated with Latinx populations. Toward that end, the Housing Instability Scale data that were collected in Spanish were compared with English responses. Rather than simply looking for group differences across items, a measurement invariance framework was used to determine if the scale functioned similarly across languages.

### Measures

Although the parent study included measures of survivor safety, mental health, substance use, housing stability, economic stability, and wellbeing, only measures used to validate the Housing Instability Scale are described here.

#### Development of Housing Instability Scale.

A seven-item Housing Instability Scale (HIS; see Table 1 for individual items in English and Spanish) was created for this study by modifying the 10-item Housing Instability Index (HII; Rollins et al., 2012). Four of the 10 HII items were removed as they related to issues with landlords, and many of the current study’s participants did not have landlords. The HII also included no measure of homelessness, so we included the item: “Have you been homeless or had to live with family or friends to avoid being homeless?” Of the seven final scale items, five included dichotomous yes/no responses while two items were re-coded to be dichotomous. Specifically, the question, “In the past 6 months, how many times have you moved?” was dichotomized and counted as a risk factor if participants reported moving more than twice in the past 6 months. “How likely is it that you will be able to pay for your housing this month?” was re-coded so that 0 represented a response of “very likely” or “somewhat likely” and 1 represented a response of “unlikely” or “very unlikely.” “Do you expect that you will be able to stay in your current housing for the next 6 months?” was reverse-coded so that a response of “no” was counted as a risk factor. For each item, then, 0 = not a risk factor and 1 = a risk factor. Sum scores could therefore range from 0 to 7, with higher scores indicating higher instability. Cronbach’s alpha for the sum score was .79 (*M* = 3.00, *SD* = 2.24).

We note that six of these seven items ask participants to respond regarding their past or projected experiences over a six-month period. This is due to the fact that the HIS conceptualizes the measurement of housing instability as the observation of phenomena occurring over a period of time, rather than simply measuring the current details of an individual’s housing situation. Therefore, it was necessary to inquire beyond participants’ present housing status.

#### Satisfaction with housing

Satisfaction with housing was measured by one item that asked “How do you feel about your current housing situation?” Responses were recorded on a 7-point scale and included: 1 = “terrible,” 2 = “unhappy,” 3 = “mostly dissatisfied,” 4 = “mixed – equally satisfied and dissatisfied,” 5 = “mostly satisfied,” 6 = “happy,” and 7 = “extremely happy.”

#### Financial strain

Financial strain was measured by the two-item Financial Strain subscale from Barrera et al.’s (2001) Scale of Economic Hardship. The two-item Financial Strain subscale measures expected future financial strain over the next 6-months (3 months in the original scale). The two questions were “How often do you think that you and your family will experience bad times such as poor housing or not having enough food?” and “How often do you expect that you will have to do without the basic things your family needs?” The original responses ranged from 1 to 5: 1 = “almost never” to 5 = “almost always.” The response options were slightly modified for the current study: 0 = “never,” 1 = “hardly ever,” 2 = “some-times,” 3 = “often,” and 4 = “quite often.”

#### Inability to make ends meet

Inability to make ends meet was measured by the two-item Inability to Make Ends Meet subscale from Barrera et al.’s (2001) Scale of Economic Hardship and refers to financial difficulty experienced over the prior 6-months (3 months in the original scale). We slightly modified the wording of the response options for difficulty paying bills (worded “how difficult has it been to pay your bills in full?”). The original options were “no difficulty at all,” “a little difficulty,” “some difficulty,” “quite a bit of difficulty,” and “a great deal of difficulty.” These options were replaced with a 4-point scale: 0 = “not at all difficult,” 1 = “a little difficult,” 2 = “somewhat difficult,” and 3 = “very difficult.” Having money left over at the end of the month was rated on the original 5-point scale: 5 = “more than enough money left,” 4 = “some money left,” 3 = “just enough money left,” 2 = “somewhat short of money,” and 1 = “very short of money.”

A measure of *financial difficulties* was created specifically for this study. Survivors responded to 10 items asking if they had had enough money in the prior six months for: food, rent/mortgage, utilities, medical expenses, transportation, social activities, and to pay debts and childcare. Responses were reported using a 4-point scale of difficulty: 0 = “not difficult at all,” 1 = “a little difficult,” 2 = “somewhat difficult,” and 3 = “very difficult.” “I do not have these bills” was also included as a response option. For scale construction, these were re-coded to 0 = “not difficult at all.” Cronbach’s alpha for the 10-item measure was .87 (*M* = 2.28; *SD* = .68).

### Data analytic plan

To examine the utility of the HIS, we examined measurement invariance, concurrent validity, and predictive validity. Specifically, we conducted a series of measurement invariance tests to determine whether the scale works the same across time points as well as across language and locality. To test measurement invariance over time, all available data were used including both Spanish and English surveys. To test for measurement invariance across language and then again with community, all available data were once again used. For measurement invariance by language, a total of 218 Spanish interviews (44 on average per wave) and 1561 English interviews (312 on average per wave). For measurement invariance by the community, a total of 764 urban community interviews (153 on average per wave) and 843 rural community interviews (169 on average per wave). Models accounted for the dependence of observations through the use of cluster robust standard errors (McNeish et al., 2017). Results were consistent whether accounting for clustering associated with wave of data collection, accounting for clustering across individuals, or not accounting for clustering at all. We, therefore, report only one set of results, accounting for clustering at the participant level.

Concurrent and predictive validity were tested using the time-invariant factor structure and finding associations between HIS factor scores and current and future related variables. Concurrent validity was evaluated by correlating factor scores of the HIS with survivors’ responses to how they felt about their current housing at the same time point. Predictive validity was evaluated by using previous time points’ factor scores of the HIS to predict measurements of financial difficulty (financial difficulty, inability to make ends meet, and financial strain) at the subsequent time point. All models were conducted in Mplus version 8 (Muthén & Muthén, 1998–2017).

## Results

### Descriptive statistics

Mean levels of the HIS sum score steadily decreased throughout the course of the study, indicating that, on average, participants were becoming, as expected, more stably housed (see Table 2). Coefficient alphas for the HIS were examined at each wave of data collection (respectively; α = 0.65, 0.72, 0.77, 0.75, and 0.78), suggesting adequate internal reliability at all waves. Mean levels of the HIS sum score were also examined based on the language the scale was administered in (English or Spanish), with English interviews reporting significantly higher levels of housing instability (*M* = 3.02, *SD* = 2.80) than Spanish interviews (*M* = 2.20, *SD* = 1.73), *t*(1729) = 5.094, *p* < 0.001, *d* = 0.37.

### Measurement invariance across time

Measurement invariance over time was tested by comparing a series of nested confirmatory factor analysis (CFA) models, each with more stringent constraints following standard procedures for measurement invariance testing (Muthén & Asparouhov, 2002). Specifically, for our configural invariance model, we fit a CFA with an autocorrelated residual structure at the item level. This model fit the data well (χ^2^ (522) = 793.646, *p* < 0.001; RMSEA = 0.036; CFI = 0.94; TLI = 0.94), indicating that a single factor model was appropriate at each wave across the study. Next, we compared the configural model to a weak measurement invariance model in which we fixed each item to have equal factor loadings at each time point. This model did not add a significant amount of misfit (Δ χ^2^(24) = 35.708, *p* = 0.059, ΔCFI = 0.003). Finally, we tested for strong measurement invariance by comparing a model with all factor loadings and thresholds constrained to be equal across time to the configural invariance model. This model fit significantly worse (Δ χ^2^(20) = 74.087, *p* <0.001) but still fit the data well (χ^2^ (542) = 849.568, *p* < 0.001; RMSEA = 0.037; CFI = 0.94; TLI = 0.93) with a small change in overall fit (ΔCFI = 0.007) which indicates that strong measurement invariance was supported (Cheung & Rensvold, 2002). To further explore this assertion, we examined modification indices to identify potential sources of misfit in the strong invariance model. After relaxing the equality constraints on the thresholds for two items (“Do you expect to stay in your current housing for the next 6 months?” and “How likely is it, do you think, that you will be able to pay for your housing (e.g. rent/mortgage) this month?”), we found evidence for partial strong invariance (Δ χ^2^(12) = 19.664, *p* = 0.074, ΔCFI < 0.001). Combined, these results suggest that the HIS is measuring the same construct, on the same scale, with the same origin across multiple waves of data collection. These results provide preliminary evidence that researchers can be confident giving this scale to participants across multiple waves of a study, and across various levels of housing stability.

### Measurement invariance by language

Once measurement invariance by time was established, we examined if there were differences in the way this construct is measured based upon whether the English or Spanish version of the scale was administered. Measurement invariance was examined by the same process described earlier (see data analytic plan). We first fit a configural model to the data, which demonstrated adequate fit (χ^2^ (28) = 78.824, *p* < 0.001; RMSEA = 0.045; CFI = 0.99; TLI = 0.99). Next, we compared that model to a weak measurement invariance model in which we fixed items to load equally onto their respective factor across language and found that this model did not add a significant amount of misfit (Δ χ^2^(6) = 11.056, *p* = 0.087, ΔCFI = 0.001). Finally, we tested for strong measurement invariance by comparing a model which fixed all factor loadings and thresholds to be equal across language to the configural model. Similar to the strong invariance across time model, this model added a significant amount of misfit (Δ χ^2^(5) = 15.913, *p* = 0.007). However, the change in fit was negligible (ΔCFI = 0.001), suggesting that the HIS has strong measurement invariance across language. To further explore this assertion, we examined modification indices to identify potential sources of misfit in the strong invariance model. Evidence of partial strong invariance (Δ χ^2^(4) = 4.852, *p* = 0.303, ΔCFI = 0.001) was found after relaxing the equality constraints on the thresholds for one item (How likely is it that you will be able to pay for your housing this month?). Again, these results suggest that the HIS reliably measures the same construct across both English and Spanish administrations. This provides preliminary evidence that future researchers can use both the English and Spanish versions of the scale in the same study and be confident that they are measuring the same construct throughout.

### Measurement invariance by locality

Finally, we examined if this scale is measuring the same construct for those who live in rural communities and those who live in urban communities. Specifically, following the same measurement invariance process described above, we first fit a configural model to the data, which demonstrated good fit (χ^2^ (28) = 123.094, *p* < 0.001; RMSEA = 0.065; CFI = 0.98; TLI = 0.98). Next, we compared that model to a weak measurement invariance model in which we fixed items to load equally onto their respective factor across community and found that this model did not add a significant amount of misfit (Δ χ^2^(6) = 8.155, *p* = 0.227, ΔCFI = 0.006). Finally, we tested for strong measurement invariance by comparing a model which fixed all factor loadings and thresholds to be equal across communities to the configural model. Similar to the weak invariance model, this model did not add a significant amount of misfit (Δ χ^2^(5) = 9.794, *p* = 0.0813, ΔCFI < 0.001). Combined, these results suggest that the HIS is measuring the same construct, on the same scale, with the same origin across both urban and rural communities.

### Concurrent validity

To assess concurrent validity, we once again modeled housing instability using the strong invariance factor structure described earlier. We then examined the correlation between the HIS factor scores (at waves 2 through 5) and participants’ feelings about their current housing situations. An individual’s HIS score was significantly associated with their feelings about their current housing situation at each of the time points (at waves 2 through 5 respectively; *r* = −0.530, *r* = −0.547, *r* = −0.519 and *r* = −0.487; all *p* < 0.001). These associations indicate that, as predicted, more housing instability was associated with less satisfaction with current housing for participants.

### Predictive validity

We next used the HIS factor at waves 1 through 4 as a predictor of three scales of financial instability measured six months later: inability to make ends meet, financial strain, and financial difficulties. Higher levels of housing instability predicted a greater inability to make ends meet six months later (*b* = .259, *p* < 0.01, *β* = 0.136), more financial strain six months later (*b* = .275, *p* < 0.001, *β* = 0.232), and more financial difficulties six months later (*b* = .216, *p* < 0.001, *β* = 0.237).

## Discussion

Given high rates of homelessness across the nation, and the deleterious consequences of housing instability (North et al., 2010; Vijayaraghavan et al., 2011; Ziliak, 2019), there is a pressing need to study both homelessness prevention and intervention efforts. The lack of a succinct scale that measures housing instability in a psychometrically reliable and valid way has hindered efforts to examine change in this construct over time. In the current study, we adapted a previous version of a succinct housing instability measure and provided evidence that this new scale is both reliable and valid. As part of a longitudinal evaluation of a housing intervention, there is evidence that the HIS reliably measures housing instability in the same way over time. As inclusion criteria required participants to be unstably housed at study entry, and baseline scores were gathered prior to engagement in the intervention, our evidence suggests that this scale is effective at measuring housing instability at various levels of instability.

Another important implication of this validation is that participants in this study were from both urban and rural areas. Most housing and homelessness research focuses predominantly on urban communities (e.g. Bottino et al., 2019; Carrion et al., 2015; etc., Desmond, 2015; Routhier, 2019). The findings from this study provide preliminary evidence of the utility of the HIS among both urban and rural individuals experiencing housing instability and/or homelessness.

This study also addresses the clear need for validated measures across multiple languages. Although some previous studies of housing instability have included Spanish-speaking immigrant Latinx samples, they have not utilized a validated measure in Spanish (Adams et al., 2021; Pavao et al., 2007). As such, there is a possibility that housing instability was not measured the same way across languages, presenting a methodological issue with important policy and practice implications. This highlights that, when working with multilingual samples, it is critical to intentionally integrate language considerations throughout the research process, including study design, data collection, and analytic procedures (López-Zerón et al., 2021). Grounded in our commitment to equity and language justice, we described relevant recruitment, translation, data collection, and validation procedures, while detailing the steps we took to ensure that the HIS measures the same construct across various levels of housing instability in both English and Spanish.

### Study limitations

Several limitations should be considered when interpreting study findings. First, it is important to recognize that this measure does not capture housing quality, nor does it provide a nuanced understanding of homelessness. That is, homelessness is considered to be the most extreme form of housing instability, and its inclusion in the scale is in recognition of the extent to which people move in and out of homelessness. This brief scale is not intended to comprehensively capture the experiences of homelessness, and we refer to Tsemberis et al.s’ (2007) timeline follow-back inventory for such a measure. As part of a larger project, the current study did not focus on distinguishing a cutoff score for housing instability. Thus, future research should explore the score on the HIS at which someone would be considered unstably housed. Further, as we had a predominantly cis-gender female sample, future studies should examine the effectiveness of the HIS in assessing housing instability among other genders (i.e. cis-gender males, trans individuals, etc.). The HIS, while limited in breadth and scope, is a reliable and valid brief measure, ideal for inclusion in studies that measure multiple constructs.

The current study also included only survivors of domestic violence, who were almost entirely cis-gender, heterosexual women. While this is an appropriate population to validate the scale on, given the link between domestic violence and housing instability (e.g. O’Campo et al., 2015; Pavao et al., 2007), additional validation measures should be undertaken with other populations and across other languages.

### Implications for policy, research, and practice

The validation of the HIS has important implications for the ongoing study of housing instability, as well as for intervention efforts aimed at mitigating the consequences of housing instability. Given practice and policy implications of housing instability and homelessness across the country, we desperately need measures that are easy to implement and analyze. The HIS fills those gaps by providing a measure of housing instability that researchers, evaluators, and community-based practitioners can easily implement in their work. Finally, we hope that future studies using the HIS can quickly and accurately provide key stakeholders and policy makers with information about housing needs in their communities.

## Conclusion

In closing, housing instability can create a myriad of detrimental impacts. As such, significant efforts have been made by social agencies and policymakers to support individuals in securing long-term stable housing. However, providing housing is only the first step in supporting these individuals in their journey to successful self-reliance. As a succinct, reliable, and valid measure of housing instability, the HIS can serve as an effective and feasible tool in assessing individuals’ progress in maintaining stable housing.

## Figures and Tables

**Table 1.** Questions and response options for the housing instability scale.

	Items in English	Items in Spanish	Response codes in English*	Response codes in Spanish*
1	In the last 6 months, have you had to live somewhere that you did not want to live?	En los últimos 6 meses, ¿ha tenido que vivir en algún lugar que no quisiera vivir?	0 = no 1 = yes	0 = no 1 = sí
2	Do you expect to stay in your current housing for the next 6 months?**	¿Espera permanecer en su vivienda actual durante los próximos 6 meses?**	0 = yes 1 = no/don’t know	0 = sí 1 = no/ no sé
3	In the last 6 months, have you been homeless or had to live with family or friends to avoid homelessness?	En los últimos 6 meses, ¿ha estado sin hogar o ha tenido que vivir con su familia o amigos para evitar quedarse sin hogar?	0 = no 1 = yes	0 = no 1 = sí
4	In the last 6 months, have you had difficulty paying (or were you unable to pay) for housing?	En los últimos 6 meses, ¿ha tenido dificultades para pagar (o no pudo pagar) por su vivienda?	0 = no 1 = yes	0 = no 1 = sí
5	In the last 6 months, have you had trouble getting housing?	En los últimos 6 meses, ¿ha tenido tenido problemas para conseguir vivienda?	0 = no 1 = yes	0 = no 1 = sí
6	How many times have you moved in the last 6 months?	¿Cuántas veces se ha mudado en los últimos 6 meses?	0 = 0–2 moves 1 = 3 or more moves	0 = 0–2 mudanzas 1 = 3 o más mudanzas
7	How likely is it, do you think, that you will be able to pay for your housing (e.g. rent/mortgage) this month?**	¿Qué tan probable cree que es que usted pueda pagar su vivienda (por ejemplo, alquiler o crédito hipotecario) este mes?**	0 = likely, very likely 1 = very unlikely, unlikely	0 = muy probable, probable 1 = muy poco probable, poco probable

* Higher scores indicate higher housing instability.

** Original response options are reverse coded.

**Table 2.** Descriptive statistics and correlations for housing instability scale scores.

Variable	*n*	*M* ^a^	*SD*	1	2	3	4	5
1. Housing Instability at Baseline	406	4.78	1.67	–				
2. Housing Instability at 6 months	373	3.42	2.04	.428**	–			
3. Housing Instability at 12 months	366	2.41	2.09	.317**	.580**	–		
4. Housing Instability at 18 months	358	2.08	1.98	.243**	.420**	.539**	–	
5. Housing Instability at 24 months	362	1.69	1.91	.235**	.324**	.383**	.557**	–

** *p* < 0.01.

a Range for mean HIS score is from 0 to 7.
